# Angiotensin II promotes erythroid proliferation in a three-stage erythroid culture system

**DOI:** 10.1016/j.mex.2024.102714

**Published:** 2024-04-12

**Authors:** Saiphon Poldee, Chanatip Metheetrairut, Wichit Suthammarak, Kongtana Trakarnsanga

**Affiliations:** Department of Biochemistry, Faculty of Medicine Siriraj Hospital, Mahidol University, Bangkok, 10700, Thailand

**Keywords:** Erythroid culture system, *In vitro* erythropoiesis, Angiotensin II, Erythroid proliferation, A 3-stage erythroid culture system with angiotensin II

## Abstract

At present, the numbers of cultured erythroid cells obtained from culture systems are not on a scale that can be used for therapeutics since the cultured erythroid cells have limited proliferation capacity. Stromal cells are believed to play important roles during erythropoiesis. Our previous study shows that factors secreted by stromal cells enhance the proliferation capacity of adult erythroid cells in the culture system. Among the identified factors, angiotensinogen is one of the most abundant proteins secreted by the stromal cells. This study aims to investigate the effect of angiotensin II, an angiotensinogen derivative, on the proliferation of erythroid cells.

• The receptor for angiotensin II was first checked by PCR analysis. It was expressed in erythroblasts at all stages during differentiation.
• To study the effect of angiotensin II, CD34^+^ hematopoietic stem cells were cultured in a 3-stage erythroid culture system with and without angiotensin II. The addition of angiotensin II to the culture media, from day 0 to 8, significantly increases the numbers of cultured erythroid cells, whereas no difference in enucleation is observed.

## Graphical abstract

**Figure:**
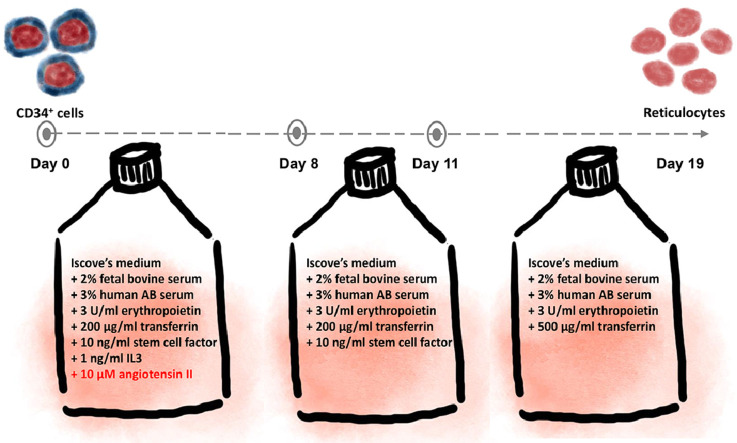

Specifications table

**Table:** 

Subject area:	Medicine and Dentistry
More specific subject area:	Regenerative medicine
Name of your method:	A 3-stage erythroid culture system with angiotensin II
Name and reference of original method:	A 3-stage erythroid culture system from: Griffiths RE, Kupzig S, Cogan N, Mankelow TJ, Betin VM, Trakarnsanga K, Massey EJ, Lane JD, Parsons SF, Anstee DJ. Maturing reticulocytes internalize plasma membrane in glycophorin A-containing vesicles that fuse with autophagosomes before exocytosis. Blood. 2012 Jun 28;119(26):6296–306. doi:10.1182/blood-2011-09-376475
Resource availability:	N/A

## Method details

In order to produce clinical quality blood products for transfusions, scientists worldwide are aiming to develop *in vitro* culture systems to generate red blood cells. Due to limited potential for proliferation, extrapolated cell counts generated now still fall short of the amount needed for therapies. Therefore, alternative approaches are needed to get past this obstacle.

It has been suggested that stromal cells are essential for erythropoiesis [1,2]. According to our earlier studies, stromal cell-secreted factors enhance adult erythroid cells' capacity to proliferate in a culture system [2]. However, the production of GMP-grade blood products does not allow the use of stromal cell co-culture or conditioned media. Therefore, it is essential to identify the specific factors that stimulate erythroid proliferation in order to improve our culture systems to raise the yield of red blood cell production for therapeutic usage. Angiotensinogen, a precursor protein for angiotensin II, is one of the main proteins released by the stromal cells [2]. Within the renin-angiotensin system (RAS), angiotensin II is the most active peptide hormone [3]. Autosomal-recessive renal tubular dysgenesis (ARRTD) is a fatal disease due to abnormal renal tubular development resulting from mutations in genes encoding proteins that operate in the RAS [4]. Because of neonatal renal and pulmonary failure, this illness is lethal and results in early death. One symptom that has been noted in individuals who have survived the neonatal stage is anemia [5]. On the other hand, renal artery stenosis with low renal blood flow triggers the RAS which further increases erythropoiesis [6]. These findings indicate a relationship between the RAS and erythropoiesis. Previous study shows that angiotensin II promotes the proliferation of murine bone marrow and human cord blood hematopoietic stem cells [7]. Moreover, an addition of erythropoietin to the culture media containing angiotensin II increases the numbers of erythroid progenitor cells developed from CD34^+^ cells [7,8,9]. It has been shown that angiotensin II also enhances the expansion of early erythroid progenitor cells [8,9]. However, the effect of angiotensin II on differentiating erythroid cells has never been explored. Therefore, the purpose of this study is to investigate how angiotensin II affects the expansion of differentiating erythroid cells.

## Materials and methods

To study the effect of angiotensin II on differentiating erythroid cells, discarded leucocyte reduction system (LRS) cones were used as a source of CD34^+^ progenitor cells. A MiniMacs direct CD34^+^ progenitor cell isolation kit (130-406-703, Miltenyi Biotech Ltd.) was used to isolate CD34^+^ cells from the blood that remained in the LRS cones following the manufacturer's procedures, which are briefly mentioned below [10].

1. About 2 ml of blood remained in each discarded LRS cone. The blood was collected and diluted by adding 2 ml of Hanks' Balanced Salt Solution (HBSS) (H6648, Sigma-Aldrich), which contained 0.6% (v/v) citrate dextrose solution (ACD) (C3821, Sigma-Aldrich). The diluted blood was layered on top of 4 ml of Ficoll-Histopaque solution (Histopaque®−1077 Hybri-Max™, H8889, Sigma-Aldrich), and centrifuged at 400 g for 30 min at 20 °C.
2. The middle interface layer containing mononuclear cells were collected and washed twice with 10 ml HBSS containing 0.6% ACD. A pre-warm red cell lysis buffer (150 mM NH_4_Cl (A0171, Sigma-Aldrich), 1 mM EDTA·2H_2_O (BDH4616, BDH Laboratory Supplies) and 10 mM KHCO_3_ (PT1195, Bio Basic), pH-adjusted to 7.5 with NaOH (S318–500, Fisher Scientific)) was then added to the cells and incubated for 12 min at 37 °C.
3. Following incubation, the cells were spun down and washed once with 10 ml HBSS containing 0.6% ACD. The cell pellet was then resuspended in cold MACs buffer (0.5% Bovine serum albumin (A8806, Sigma-Aldrich), 0.6% Citrate phosphate dextrose (CPD) (C7165, Sigma-Aldrich) in 1x PBS (D1408, Sigma-Aldrich)) and centrifuged at 400 g for 5 min at 20 °C.
4. Following a wash with MACs buffer, the cells were incubated in 500 µl cold MACs buffer that contained 100 µl of MACS-Fc blocking agent and 100 µl of MACS-CD34 beads for 30 min at 4 °C while being constantly mixed using a roller.
5. After a single washing in 5 ml of cold MACs buffer, the cell pellet was again resuspended in 3 ml of cold MACs buffer. The cell suspension was applied to a pre-wet LS column. After three rounds of washing the column with 1.5 ml of cold MACS buffer, the cells were eluted using 2 ml of cold MACS buffer. The cell suspension was applied to a pre-wet MS column. The column was washed with 0.5 ml cold MACS buffer for 3 times and the CD34^+^cells were then eluted with 1 ml cold MACS buffer.

## Erythroid differentiation of CD34^+^ cells

The three-stage erythroid culture method outlined by Poldee et al. [10] was used to culture the CD34^+^ cells. For the first 8 days, the cells were cultured in Basic medium which was Iscove's medium (FG0465, Biochrom) containing 3% (v/v) human AB serum (H4522, Sigma-Aldrich), 2% fetal calf serum (Hyclone, SH30070.03 Fisher Scientific, Ltd), 3 U/ml EPO (Roche), 200 µg/ml transferrin (2914-HT, R&D Systems) and 1 U/ml penicillin/streptomycin (P4333, Sigma-Aldrich) supplemented with 10 ng/ml SCF (255-SC-050, R&D Systems) and 1 ng/ml IL-3 (233-IL-010, R&D Systems). From day 8 onward, the culture medium was adjusted by removing IL-3. Furthermore, from day 11 onward, the culture medium was further modified by removing SCF and increasing the concentration of transferrin to 500 µg/ml. Angiotensin II (A9525, Sigma-Aldrich) was added to the cultured media as indicated. Every 2–3 days, the numbers of cells were evaluated and fresh medium at the appropriate stage was added into the culture. Cell density was kept at 1–2 × 10^5^ cells/ml, 2–5 × 10^5^ cells/ml and 1–2 × 10^6^ cells/ml for days 0–8, days 8–11 and days 11–19, respectively. Total medium changes were performed on day 8 and 11 when the types of media were switched. Cells were maintained at 37 °C, 5% CO_2_. On day 19, morphological analysis was performed using cytospin and Leishman staining. The variations in cell counts and enucleation rates between the control and angiotensin II treated groups were statistically assessed using a two-sample equal variance *t*-test.

## Polymerase chain reaction (PCR)

PCR analysis was utilized to verify the expression of the angiotensin II receptor type I (AT1). Aliquots of cells were taken at specified time points throughout the culture period. RNA was extracted from cells using Trizol® reagent (15596026, Thermo Fisher Scientific). Total RNA was converted to cDNA and the AT1 sequence was amplified using primers [8] as shown in Table 1.

**Table 1:** AT1 primers.

AT1-FW	5 -tttagcactggctgacttatg- 3
AT1-RV	5 -agaaaaggaaacaggaaaccca- 3
nested primer AT1-FW	5 -ttactgactttgccactatg- 3
nested primer AT1-RV	5 -cataatggaaagcacaaact- 3.

## Results

**Fig. 1:**
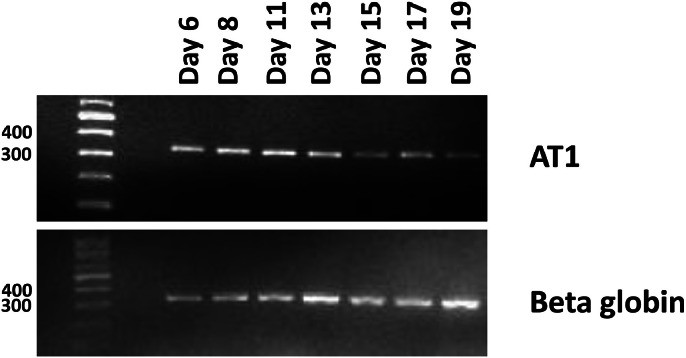
**Expression of the angiotensin receptor (AT1) during erythroid differentiation.** Erythroblasts were cultured in a 3-stage culture system, and aliquots of cells were collected at the indicated time points for RNA isolation, reverse transcription and PCR analysis. Beta globin was used as control.

Initially, as the angiotensin II receptor type I (AT1) was mentioned to play important role in adult mammals [3], we utilized PCR analysis to verify the presence of its expression in the cultured erythroblasts. Similar to the previous studies, the AT1 is expressed on hematopoietic stem cells [7,8] throughout the whole erythroid differentiation process [8] (Fig. 1).

**Fig. 2:**
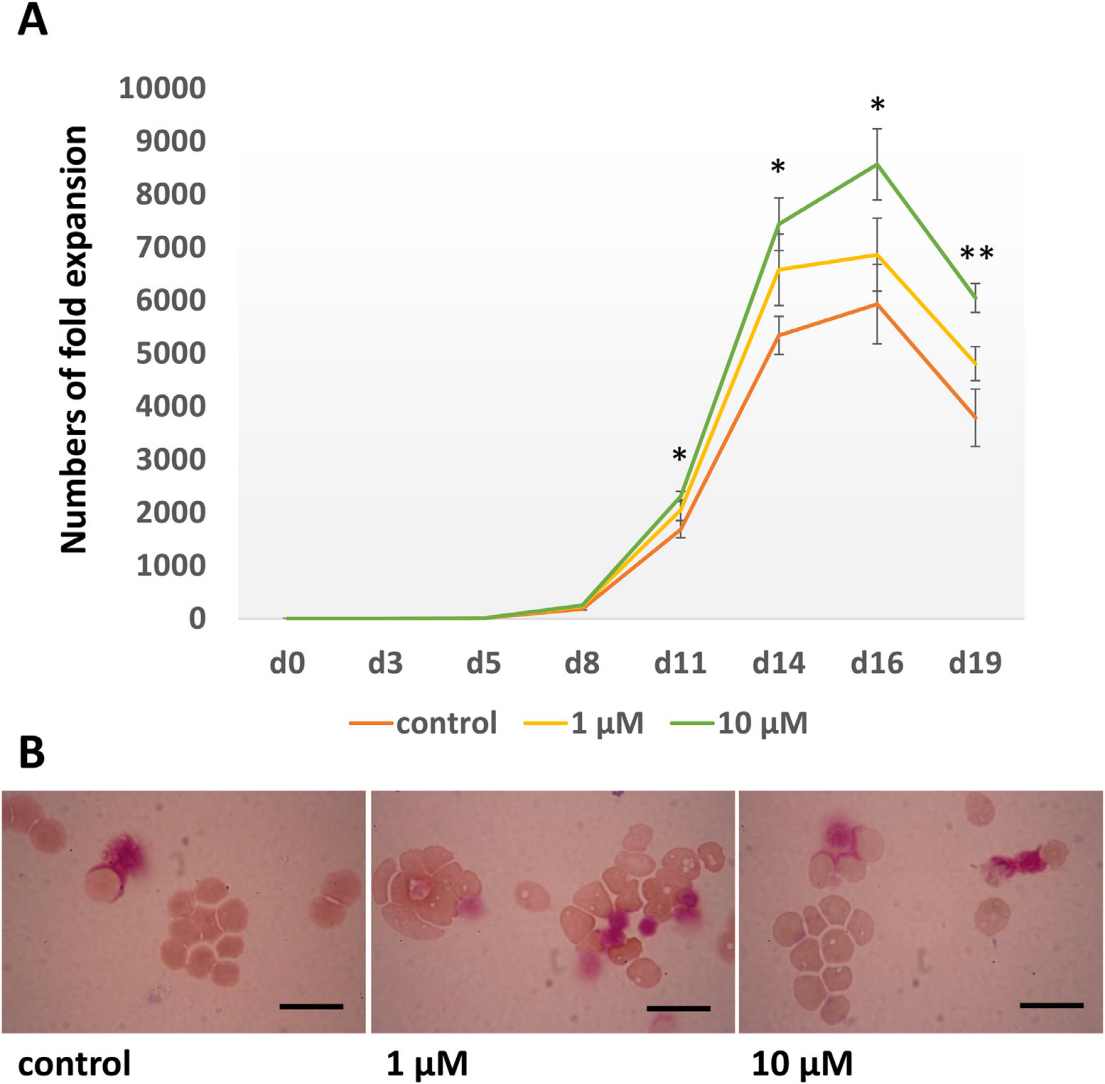
Erythroid cells maintained in a 3-stage culture system without (control) and with 1 and 10 µM angiotensin (A) Numbers of fold expansion of untreated control culture (orange), 1 µM angiotensin treated culture (yellow) and 10 µM angiotensin treated culture (green) (mean ± SD, *p < 0.05 between control and 10 µM angiotensin treated cultures, **p < 0.01 between control and 10 µM angiotensin treated cultures, by T-test, *n* = 3). (B) Morphology of cells at day 19. Cultured cells were stained with Leishman reagent and analysed by a light microscopy (scale bars 20 µm).

To study the effect of angiotensin II on erythroblast proliferation, 10^4^ CD34^+^ cells were cultured through the 3-stage erythroid culture system supplemented with 1 µM or 10 µM angiotensin. A group with no angiotensin II supplemented was also included as a control. The numbers of erythroid cells were assessed every 2–3 days. The number of cultured erythroid cells was significantly higher in the group treated with 10 µM angiotensin II than in the control group on days 11, 14, 16 and 19 (Fig. 2A). However, there was no significant difference in cell numbers between the 1 µM angiotensin-treated group and the control group. In addition, aliquots of cells were also collected on day 19 for staining for morphological analysis. There was no difference in differentiation between cells cultured in media with and without angiotensin II with the enucleation rates at day 19 of 95.0 ± 1.0%, 96.3 ± 1.2% and 94.8 ± 1.0% for 1 µM, 10 µM angiotensin II treated and control cultures, respectively (*n* = 3) (Fig. 2B). Since 10 µM angiotensin II enhances the proliferation of erythroid cells in the culture system, this concentration was selected to be used in later experiments.

**Fig. 3:**
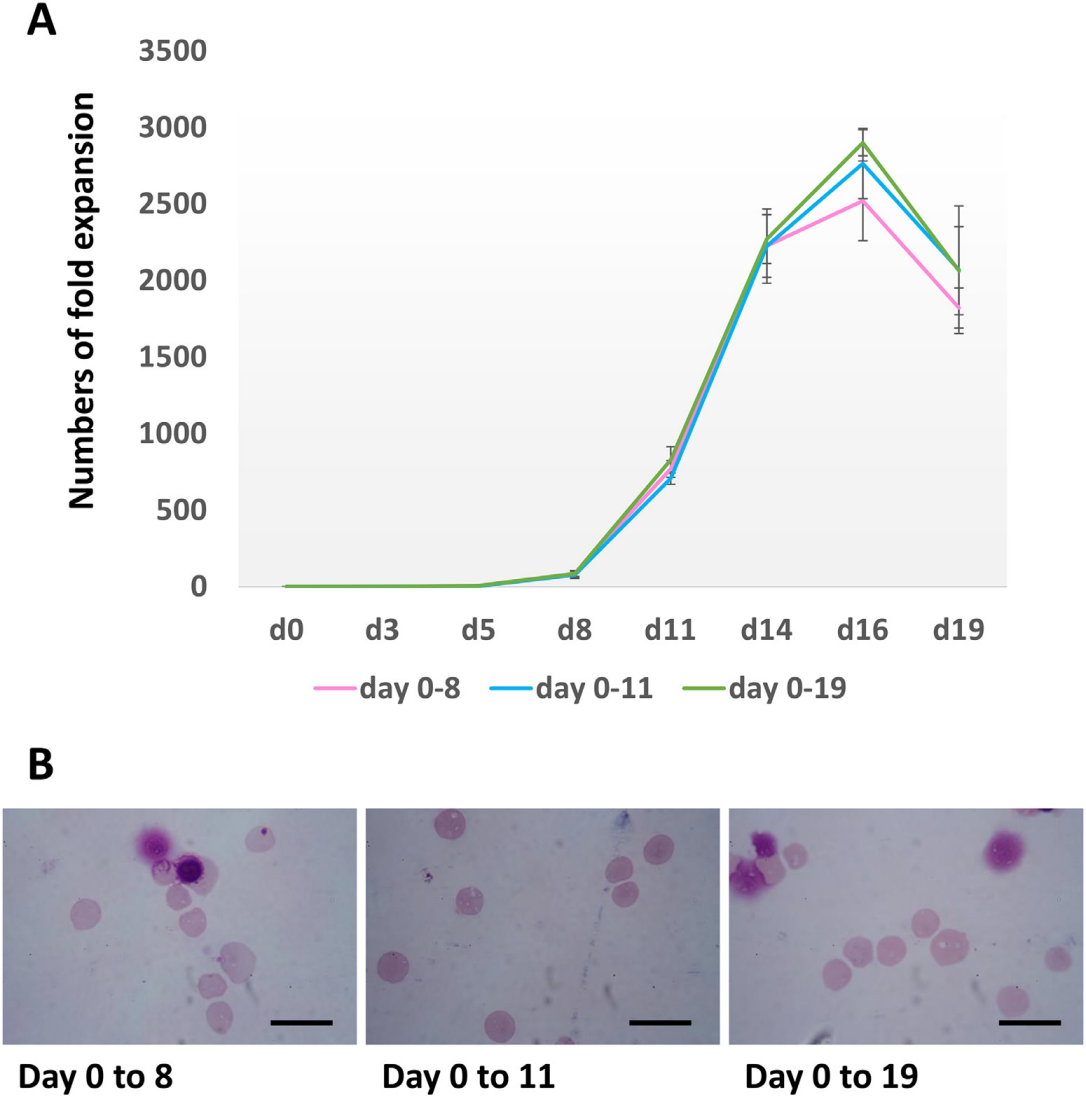
Erythroid cells maintained in a 3-stage culture system with 10 µM angiotensin added from day 0–8, day 0–11 or day 0–19 (A) Numbers of fold expansion of 10 µM angiotensin treated cultures, from day 0 to 8 (pink), from day 0 to 11 (blue) and from day 0 to 19 (green) (mean ± SD, by T-test, *n* = 3). (B) Morphology of cells at day 19. Cultured cells were stained with Leishman reagent and analysed by a light microscopy (scale bars 20 µm).

Next, we investigated whether angiotensin II was required throughout the culture period. Aliquots of 10^4^ CD34^+^ cells were cultured through the 3-stage erythroid culture system. Three groups of cultured cells were included in this experiment. Ten µM angiotensin II was added to the culture media throughout the culture period for the first group as a control. Ten µM angiotensin II was added to the culture media from day 0 to day 8 and day 0 to day 11 for the second and third groups, respectively. The numbers of erythroid cells were assessed every 2–3 days. There was no significant difference in cell numbers among all groups (Fig. 3A). Moreover, aliquots of cells were also collected for staining for morphological analysis on day 19, there was also no difference in enucleation rates among the three groups (Fig. 3B). The enucleation rates were 94.8 ± 1.4%, 94.7 ± 0.6% and 94.7 ± 0.7% for the 1st, 2nd and 3rd groups, respectively (*n* = 3). This result demonstrates that angiotensin II is required for only the early erythroid differentiation stage.

**Fig. 4:**
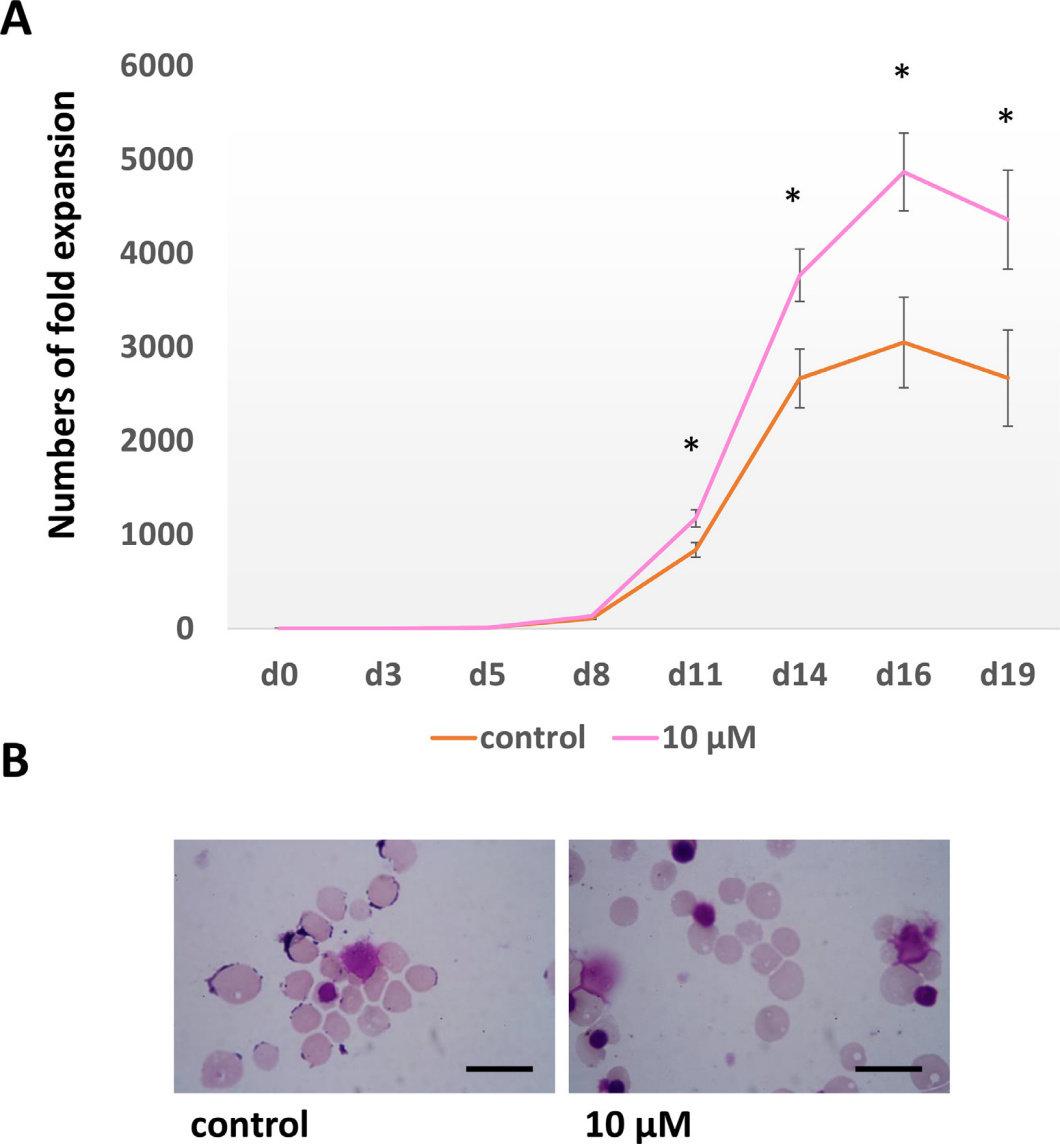
Erythroid cells maintained in a 3-stage culture system with 10 µM angiotensin added from day 0–8 and without angiotensin as control (A) Numbers of fold expansion of untreated control culture (orange), and 10 µM angiotensin treated from day 0 to 8 culture (pink) (mean ± SD, *p < 0.05 between control and 10 µM angiotensin treated cultures, by T-test, *n* = 3). (B) Morphology of cells at day 19. Cultured cells were stained with Leishman reagent and analysed by a light microscopy (scale bars 20 µm).

To confirm that the addition of angiotensin II is required for only the early differentiation stage to promote erythroid proliferation. Aliquots of 10^4^ CD34^+^ cells were cultured through the 3-stage erythroid culture system with 10 µM angiotensin II added to the culture medium from day 0 to day 8 compared to another culture with no angiotensin II added to the media as control. As expected, the numbers of cultured erythroid cells were significantly higher in the group treated with 10 µM angiotensin II from day 0 to day 8 than in the control group on days 11, 14, 16 and 19 (Fig. 4A). Aliquots of cells were also collected for staining for morphological analysis on day 19 (Fig. 4B). There was no difference in enucleation rates between the 10 µM angiotensin II treated culture (94.6 ± 0.7%) and the control culture (93.7 ± 3.9%) (*n* = 3).

In this study, we show that angiotensin II significantly increases the proliferative potential of human adult differentiating erythroid cells when it was added to the culture medium from day 0 to day 8. The number of cultured reticulocytes obtained from cultures with addition of angiotensin II was nearly 2-fold higher than those using the control medium. This improvement brings the numbers of *in vitro* generated red blood cells closer to those required for therapy.

## Ethics statements

Leucocyte reduction system (LRS) cones were obtained from healthy donors with written informed consent for research use in accordance with the Declaration of Helsinki. This protocol (SIRB Protocol number 438/2559) complies with a “research with exemption” category by the Siriraj Institutional Review Board.

## Supplementary material *and/or* additional information [OPTIONAL]

N/A.

## CRediT authorship contribution statement

**Saiphon Poldee:** Methodology, Validation, Formal analysis, Investigation, Visualization, Project administration. **Chanatip Metheetrairut:** Conceptualization, Formal analysis, Writing – review & editing. **Wichit Suthammarak:** Conceptualization, Formal analysis, Writing – review & editing. **Kongtana Trakarnsanga:** Conceptualization, Methodology, Validation, Formal analysis, Investigation, Visualization, Writing – original draft, Supervision, Funding acquisition.

## Declaration of competing interest

The authors declare that they have no known competing financial interests or personal relationships that could have appeared to influence the work reported in this paper.

## Data Availability

No data was used for the research described in the article.
